# Immediate Effects of Brief Exposure to the Healthy Eating Plate on Adults’ Nutrition Knowledge: A Cross-Sectional Survey

**DOI:** 10.3390/nu18040697

**Published:** 2026-02-21

**Authors:** Justyna Malinowska, Magdalena Jodkiewicz, Karolina Marek-Woźny

**Affiliations:** Medical Center of Dietetics and Health Education, National Institute of Public Health NIH-National Research Institute, Chocimska 24, 00-791 Warsaw, Poland; m.jodkiewicz@gmail.com (M.J.); kmarek-wozny@pzh.gov.pl (K.M.-W.)

**Keywords:** nutrition education, Polish healthy eating plate, dietary recommendations, pre-post survey study

## Abstract

Introduction: The Healthy Eating Plate is a graphical presentation of Polish healthy eating recommendations introduced in 2020. This study evaluated the extent to which the model and its accompanying materials improve adults’ short-term recall and comprehension of healthy eating principles. Materials and Methods: A cross-sectional online survey was conducted in Poland (19–30 September 2025) using the MNForce Poland research panel. The sample comprised 200 adults aged 18–65 years. Participants completed an author-developed questionnaire including demographics, a pretest, exposure to the Healthy Eating Plate and the “In 3 Steps to Health” material, an immediate post-test aligned thematically with the pretest, and items evaluating perceptions of the Healthy Eating Plate. Results: The overall knowledge index increased from 64.3% (SD 17.6) pre-exposure to 81.0% (SD 19.4) post-exposure, representing a 16.7 percentage-point improvement. This increase in short-term knowledge scores was statistically significant. The largest residual knowledge deficit concerned identifying the components of a balanced meal (38.0% correct post-test). The highest post-test performance was observed for recommendations on increasing whole-grain intake and reducing salt consumption to 5 g/day (both 91.5%). Baseline knowledge was associated with prior use of dietetic services and with self-assessed knowledge. Conclusions: Exposure to the Healthy Eating Plate and accompanying materials resulted in significant immediate improvements in recall and comprehension of healthy eating recommendations. These findings reflect short-term knowledge transfer under real-world dissemination conditions and should not be interpreted as evidence of sustained learning or behavioural impact, while highlighting the need to strengthen communication of the balanced-meal concept.

## 1. Introduction

Nutrition education can be delivered in a range of formats, including consultations with a dietitian or primary care physician, structured training courses, and the use of educational materials or programmes. The effectiveness of dietetic counselling in particular has been examined in numerous studies and was supported in a systematic review published in 2024 [1]. A small Polish study (n = 32) likewise reported a statistically significant increase in students’ nutrition knowledge following completion of a human nutrition course [2]. Evaluations of the MyPlate model and the “half-plate” principle have also yielded favourable findings, indicating that visual representations of healthy eating guidelines are generally well understood and, according to participants, may facilitate improvements in dietary habits [3]. A variety of graphical models for presenting healthy eating guidelines are currently used worldwide, differing in scope and level of detail [4]. One such model is the Polish Healthy Eating Plate [5], which has not yet been assessed in terms of comprehensibility and effectiveness in communicating healthy eating guidance.

**Figure 1:**
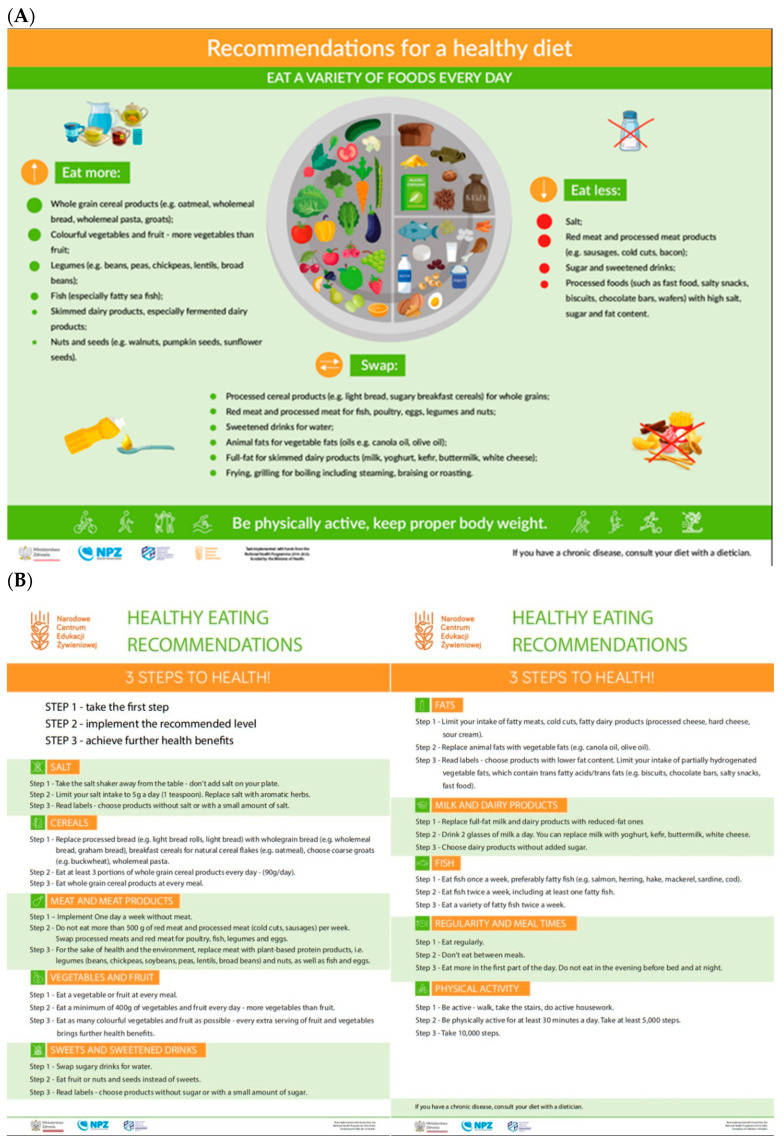
(**A**) Polish Healthy Eating Plate, and (**B**) Healthy Eating Recommendations—3 Steps to Health [5].

The Healthy Eating Plate (Figure 1A) is a graphical representation of current healthy eating guidelines, which replaced the Healthy Eating Pyramid for adults in Poland in 2020. It illustrates the recommended proportions of individual food groups in the overall daily diet. The Healthy Eating Plate graphic is complemented by written guidance—“Eat less,” “Eat more,” and “Swap”—which lists specific food groups, as well as a recommendation regarding physical activity. An additional educational resource, “In 3 Steps to Health” (Figure 1B), provides specific dietary recommendations for different food groups across three levels, enabling gradual, incremental changes in eating habits.

The format used to present the healthy eating guidelines was revised to facilitate interpretation and practical implementation. The updated recommendations were developed on the basis of a comprehensive review of scientific evidence on the effects of diet on human health, encompassing both dietary patterns and specific nutrients relevant to health outcomes. They also drew on current recommendations issued by international and national scientific societies in the fields of nutrition and public health. However, the effectiveness of the Healthy Eating Plate as an educational tool has not previously been evaluated. Assessing the extent to which the current version of the healthy eating guidelines is understandable and effective in knowledge transfer is particularly important in the context of the growing burden of overweight and obesity in Poland. According to a 2022 report by the Supreme Audit Office, 65.6% of adult Poles were affected by overweight or obesity [6].

The aim of the study was to assess the extent to which the Healthy Eating Plate graphic, together with its accompanying materials, fulfils its role in conveying knowledge about the principles of healthy eating. The study employed an author-developed survey questionnaire, which also accounted for the potential influence of selected factors such as respondents’ sex, age, educational attainment, and place of residence.

Participants’ nutrition knowledge was compared before and after exposure to the Healthy Eating Plate graphic and the accompanying materials in a randomly selected sample of adult Poles. Participants were randomly selected from the MNForce Poland panel database using stratified sampling with predefined quotas for sex, age, education level, and place of residence to approximate the structure of the adult Polish population within the study assumptions. Eligible panel members meeting inclusion criteria were randomly invited until the target quotas (n = 200) were achieved.

## 2. Materials and Methods

### 2.1. Survey Questionnaire

The study was conducted using a quantitative methodology. For the purposes of this research, a survey technique was employed, with a structured questionnaire serving as the research instrument. In collaboration with a research agency, an author-developed questionnaire was designed for this study and consisted of five sections:

1. Demographic items.
2. Pretest.
3. Presentation of the Healthy Eating Plate.
4. Post-test.
5. Questions eliciting respondents’ opinions on the Healthy Eating Plate.

Respondents were asked about their age, sex, place of residence, and educational attainment. They were also asked to self-assess their knowledge of healthy eating and to report whether they had ever used dietetic services.

The pretest comprised 11 closed-ended single-choice questions intended to assess respondents’ baseline knowledge of healthy eating principles. The subsequent section presented the Healthy Eating Plate graphic together with the supplementary material “In 3 Steps to Health.” Participants were instructed to review the educational material carefully, with the time allocated for this task limited to 5 min. A quality-control procedure was planned for this section: participants who spent ≤1 min reviewing the material were to be excluded from the analysis, as such a short review time could indicate insufficient exposure to the educational content. However, no participants met this criterion, so no exclusions were made on this basis.

The post-test consisted of 11 closed-ended single-choice questions corresponding thematically to those in the pretest. The post-test relied predominantly on true/false items to probe the same knowledge domains as the pre-test while avoiding item repetition; this design choice was intentional, although the dichotomous format entails a 50% chance-level of correct responding, which may upwardly bias post-exposure scores and apparent knowledge gains relative to more discriminating item formats.

The final section included questions asking respondents to evaluate the Healthy Eating Plate graphic in terms of comprehensibility, clarity, credibility, and attractiveness. Respondents were also asked to indicate the perceived strengths of the Healthy Eating Plate and to identify elements that, in their view, required improvement.

### 2.2. Questionnaire Development and Validation

The questionnaire was developed by the authors for the purposes of this pragmatic pre–post study assessing immediate knowledge transfer related to the Healthy Eating Plate. Item content was derived from the educational materials and each knowledge item was mapped to specific recommendations to ensure coverage and alignment with the intervention content. To support face and content validity, the draft instrument underwent an internal review by a multidisciplinary team comprising dietitians, a psychodietitian, a psychologist, and a physiotherapist, who evaluated item clarity, relevance, and consistency with the intended constructs; minor wording refinements were introduced based on this review. In addition, questions were partially adapted from the validated KomPAN questionnaire (beliefs-related section), which has been used previously in Polish populations. No pilot testing (e.g., cognitive interviews or field pretest) was conducted prior to data collection; therefore, despite the expert review and partial use of a validated source instrument, formal psychometric evaluation of the full questionnaire (e.g., test–retest reliability or item-level discrimination) was beyond the scope of this study and should be addressed in future research.

The knowledge index was constructed as a criterion-referenced, multi-domain test sampling distinct components of the dietary guidelines rather than as a unidimensional psychometric scale; therefore, internal-consistency coefficients (e.g., Cronbach’s alpha), which primarily reflect item intercorrelation, were not treated as the primary indicator of measurement quality and were interpreted with caution.

### 2.3. Study Design and Procedure

**Figure 2:**
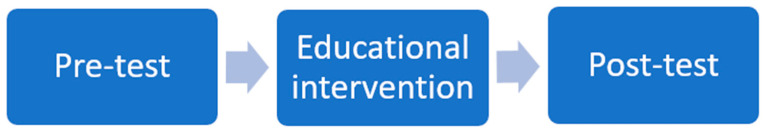
Study design flowchart.

The study design involved one group of participants who underwent a knowledge test (pretest), followed by an educational intervention (exposure to the Healthy Eating Plate) and finally a repeat knowledge test (post-test) (Figure 2). No control group was included in the study.

The study was conducted between 19 and 30 September 2025 using the MNForce Poland online research panel.

The survey was carried out on a sample of 200 adults from the Polish population aged 18–65 years. To examine the relationship between baseline nutrition knowledge and the degree of comprehension of the educational material in the context of selected factors, the sample was designed according to the following assumptions:

- Women were to constitute 50% of all respondents;
- Individuals aged 40 years and older were to account for approximately half of the sample;
- Participants with higher education (bachelor’s, engineer’s, or master’s degree) were to comprise 40–60% of the sample;
- Likewise, residents of cities with more than 100,000 inhabitants were to comprise 40–60% of the sample.

Given the study topic, individuals employed in medical professions were excluded from participation.

A control group was not included, as the study aimed to assess immediate knowledge transfer under real-world dissemination conditions, rather than to quantify causal effects within an experimental framework.

### 2.4. Sample Size Justification

No a priori power calculation was conducted because the study was designed as a pragmatic pre–post assessment of immediate knowledge transfer rather than a confirmatory effectiveness trial. The target sample size (n = 200) was selected to balance feasibility with adequate precision for item-level estimates and subgroup description across key sociodemographic strata. For proportions, n = 200 provides an approximate maximum 95% margin of error of ±7 percentage points (at *p* = 0.50), which was deemed sufficient for an initial evaluation of comprehensibility and short-term recall in an adult panel sample.

### 2.5. Statistical Analysis

Statistical analysis was performed using Microsoft Excel for Microsoft 365 MSO (Version 2304 Build 16.0.16327.20262). A *p*-value < 0.05 was considered as statistically significant.

## 3. Results

### 3.1. Characteristics of the Study Population

**Figure 3:**
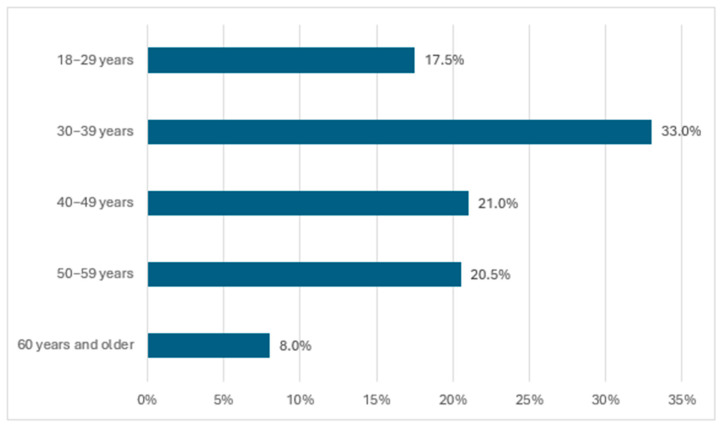
Age of the respondents (n = 200).

A total of 200 adults participated in the study. The largest age group comprised participants aged 30–39 years, and overall, 50.5% of the sample was younger than 40 years (Figure 3).

A balanced sex distribution was achieved among study participants. Both women and men accounted for exactly 50.0% of all respondents.

Respondents were drawn from a range of settlement types, allowing for a diversified perspective on the study findings. The largest proportion resided in cities with 100,000–499,999 inhabitants (27.0% of the sample). The next most numerous groups comprised residents of cities with more than 500,000 inhabitants (22.5%) and cities with 20,000–99,999 inhabitants (19.0%). Rural residents accounted for 19.5% of respondents, whereas the smallest group consisted of participants from small towns with up to 19,999 inhabitants, representing 12.0% of the sample.

Participants’ educational attainment was heterogeneous; however, individuals with higher education predominated, accounting for half (50.0%) of all respondents. The second-largest group comprised participants with general secondary or technical secondary education (30.0%). Basic vocational education was reported by 13.5% of respondents, whereas 5.0% had lower secondary education. The smallest group consisted of participants with primary education, representing 1.5% of the sample.

Respondents also assessed their level of knowledge regarding healthy eating. The largest proportion indicated that their knowledge was at a good level, with 52.0% selecting this response. A further 33.0% rated their knowledge as neither good nor poor, which may suggest a moderate level of confidence in this area. A relatively small share of participants reported very good knowledge (8.5%). Conversely, 3.0% of respondents described their knowledge as poor, and 3.5% as very poor.

Most respondents had not previously used dietetic services, as indicated by 63.0% of participants. In contrast, just over one-third of respondents (37.0%) reported having consulted a dietitian and having experience with this type of service.

### 3.2. Pretest Results

Participants in the study completed knowledge tests on healthy eating. The first of these tests was administered prior to participants’ exposure to the graphic depicting the “Healthy Eating Plate” and comprised 11 single-choice questions.

In the first test item, respondents were asked to identify the components of a balanced meal. The largest proportion (43.5%) incorrectly indicated that all of the listed options were correct. The correct response—vegetables or fruit, a protein source, grain products, and fat—was selected by 26.0% of participants. A total of 2.0% of respondents reported that they did not know the answer.

Nearly half of respondents (49.5%) incorrectly indicated that whole-grain products need only be consumed once per day. The correct answer was selected by 26.0% of participants, while the remaining 24.5% reported that they did not know the answer to this question.

The majority of respondents (76.0%) correctly agreed with the statement that a healthy diet is characterized by a higher intake of whole-grain products than refined ones. An incorrect response was selected by 7.5% of participants, while the remaining 16.5% explicitly reported that they did not know.

The vast majority of respondents (82.5%) agreed with the recommendation to replace red meat with other protein sources. By contrast, 9.5% of participants incorrectly considered this statement to be false.

As many as 85.0% of respondents correctly agreed with the statement that vegetables and/or fruit should be included in every meal. This statement was rejected by 12.5% of participants, while the remaining 2.5% selected the “I do not know” option.

The vast majority of participants (83.5%) correctly confirmed that fish—particularly fatty marine species—constitute an important component of a healthy diet. An incorrect response was selected by 8.0% of respondents, while 8.5% chose the “I do not know” option.

Nearly three-quarters of participants (73.0%) correctly identified as false the statement that only children and adolescents should consume milk. By contrast, 12.5% of respondents incorrectly judged this statement to be true.

More than half of participants (57.0%) correctly identified as true the recommendation to increase the intake of low-fat milk and dairy products, particularly fermented ones. An incorrect response was selected by 14.0% of respondents, while as many as 29.0% chose the “I do not know” option.

A total of 82.5% of respondents agreed with the statement that increasing the proportion of legumes in the diet is beneficial to health. An incorrect response was selected by 7.5% of participants, while 10.0% chose the “I do not know” option.

More than half of participants (51.5%) correctly identified as false the statement that it is necessary to completely eliminate salt intake. A substantial proportion of respondents (35.0%) selected an incorrect answer.

In the study, 64.0% of respondents knew that canola oil is a healthy product worth using in meal preparation. An incorrect response was selected by 21.5% of participants, while 14.5% chose the “I do not know” option.

Table 1 presents a consolidated comparison of the proportions of correct responses to individual questions assessing participants’ knowledge of healthy eating, which enabled comparisons across specific knowledge domains. The poorest performance was observed for questions concerning the composition of a balanced meal and recommendations regarding the frequency of consuming whole-grain products (the proportion of correct responses in both domains was 26.0%). In contrast, participants most frequently demonstrated awareness that vegetables and/or fruit should be consumed with every meal (85.0%); that fish are an important component of a healthy diet (83.5%); that red meat and processed meat products should be replaced with fish, poultry, eggs, legumes, and nuts (82.5%); and that an increased proportion of legumes in the diet is beneficial to health (82.5%).

**Figure 4:**
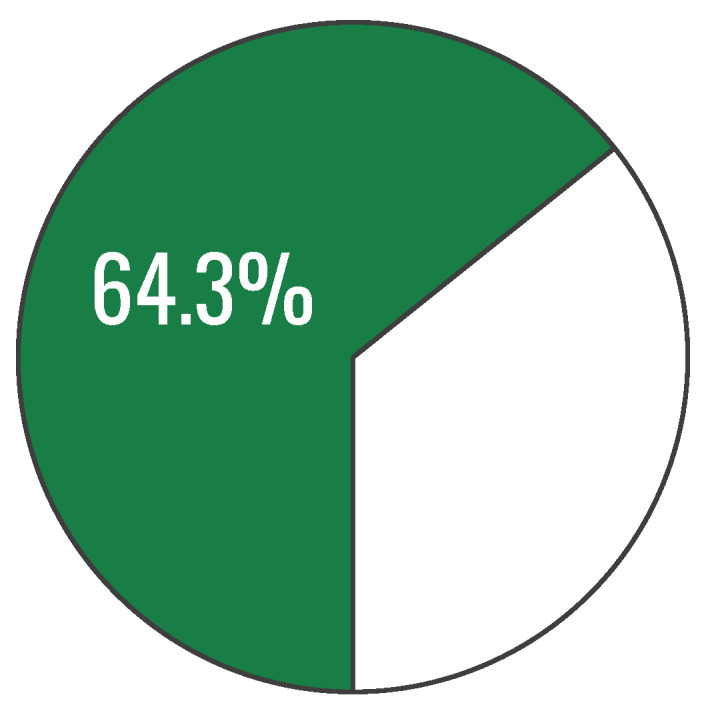
Overall index of knowledge on healthy eating (prior to exposure to the Healthy Eating Plate graphic): percentage of correct answers to the test questions (n = 200).

During the analysis of the collected research material, composite indicators aggregating responses to all knowledge-test questions were also constructed, resulting in a measure reflecting participants’ level of knowledge on healthy eating. This indicator was defined simply as the percentage of correct answers to the test questions. Its value was 64.3%. However, participants’ results were fairly heterogeneous, as evidenced by the presence in the sample of both individuals with complete knowledge of healthy eating and those with a score of zero. The standard deviation (SD) was also relatively high (17.6%) (Figure 4).

### 3.3. Post-Test Results

The study design assumed that, following the first wave of knowledge-test questions, respondents would review the Healthy Eating Plate graphic. They were then presented with a second block of test questions.

The first item in this block repeated the opening question from the previous section and therefore concerned the composition of a balanced meal. This time, the correct response (“Vegetables or fruit, a protein source, wholegrain products, and fat”) received the highest proportion of responses (38.0%). Thus, a 12-percentage-point increase in knowledge in this area was observed after exposure to the “Healthy Eating Plate” graphic. Nevertheless, a still substantial share of participants (35.5%) believed that all of the provided answers were correct.

As many as 91.5% of respondents correctly agreed with the statement recommending a higher intake of whole-grain products. An incorrect response was selected by 5.5% of participants, while the “I do not know” option received the fewest responses (3.0%).

The majority of respondents (70.5%) correctly indicated that consuming at least three servings of whole-grain products per day is recommended. An incorrect response stating that five servings are recommended was selected by 13.5% of participants, while 9.0% reported that they did not know the answer.

In response to the question concerning the recommendation to consume at least 400 g of colourful vegetables and fruit per day as an important component of a healthy diet, 88.5% of respondents provided the correct answer. An incorrect response was selected by 6.5% of participants.

As many as 81.0% of participants correctly confirmed that vegetables should account for a greater share of the diet than fruit, whereas 10.0% of respondents incorrectly considered this statement to be false.

Almost all respondents (91.5%) correctly agreed with the recommendation to reduce salt intake to 5 g per day. An incorrect negative response was selected by 5.0% of participants.

The vast majority of respondents (89.5%) correctly agreed with the recommendation to replace animal-derived fats with plant-based fats. An incorrect response was selected by 6.5% of participants.

In the study, 87.5% of respondents correctly confirmed the need to limit the consumption of red meat and processed meat products to 500 g per week. By contrast, 8.0% of participants incorrectly considered this statement to be false.

A high proportion of participants (87.0%) correctly agreed with the recommendation to consume a variety of fatty fish twice per week. An incorrect response was selected by 7.0% of respondents.

Similarly, the vast majority of respondents (86.5%) correctly confirmed the recommendation to replace meat with plant-based protein products. An incorrect response was selected by 8.0% of participants.

The final test question concerned the recommended daily serving of dairy products. A total of 80.0% of respondents correctly agreed with the statement regarding the recommended daily portion of dairy (two glasses of milk/yoghurt/kefir/buttermilk or a portion of cottage cheese). In contrast, 12.5% of participants incorrectly considered this information to be false.

A comparison of the proportions of correct responses in the post-test on healthy eating knowledge indicated that the largest deficits concerned knowledge of the composition of a balanced meal. The related item yielded only 38.0% correct responses. This may partly reflect the fact that it was the most complex question, with the largest number of response options provided. On the other hand, it was also the only question repeated from the pretest.

In the remaining test items, correct answers were endorsed by a majority of respondents. The highest proportions were observed for the questions concerning the recommendation to increase consumption of whole-grain products (91.5%) and the recommendation to reduce salt intake (91.5%) (Table 1).

**Figure 5:**
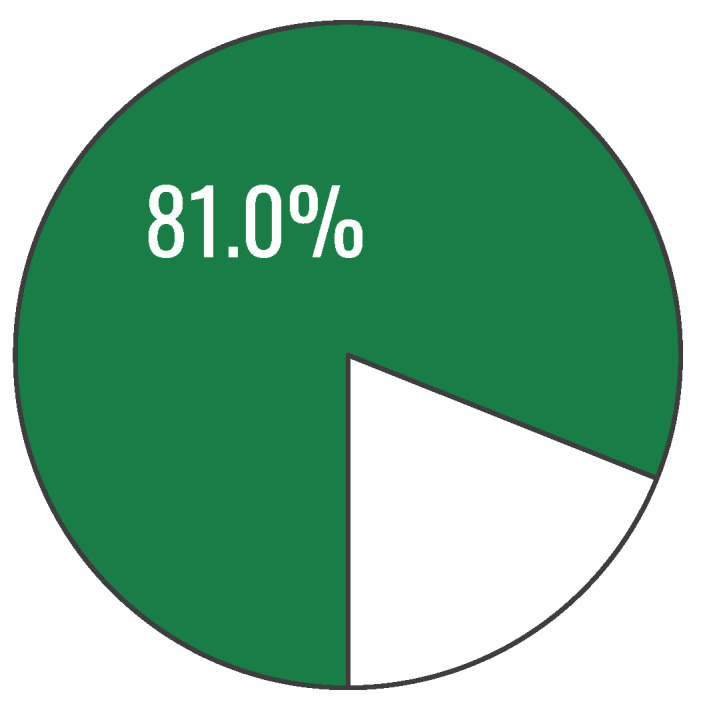
Overall index of knowledge on healthy eating (after exposure to the Healthy Eating Plate graphic): percentage of correct answers to the test questions (n = 200).

The next figure presents the value of the overall index of healthy eating knowledge after exposure to the “Healthy Eating Plate” graphic. The mean score increased from 64.27% (SD = 17.59 percentage points) at pre-test to 81.05% (SD = 19.44 percentage points) at post-test, corresponding to a mean improvement of 16.77 percentage points; a paired-samples t test indicated a statistically significant difference, t(199) = −12.19, *p* = 6.95 × 10^−26^ (two-tailed), with a 95% CI for the mean improvement of [14.06, 19.49] percentage points and a large effect size (d_x_ = 0.86) (Figure 5).

### 3.4. Respondents’ Evaluation of the Healthy Eating Plate Graphic

The study also examined participants’ opinions of the Healthy Eating Plate graphic. As an initial step, respondents were asked whether they had previously encountered this graphic. An affirmative response was reported by 36.5% of participants. Thus, the reach of this material can be assumed to have been slightly above one-third of the sample.

Respondents were also asked to evaluate the Healthy Eating Plate graphic. The item comprised eight pairs of opposing descriptors (e.g., incomprehensible–comprehensible, boring–interesting), which participants rated using a 7-point semantic differential scale. Mean ratings for all assessed dimensions were above the midpoint of the scale, indicating an overall positive reception of the visual material. In particular, the graphic was perceived as comprehensible, clear, credible, interesting, attractive, and convincing (mean scores > 5). It was also rated as motivating and as containing an appropriate amount of information (mean scores > 4.90). The analysis further examined whether demographic characteristics influenced perceptions of the graphic; however, no evidence was found that any of these variables correlated with the responses reported.

The survey questionnaire also included two open-ended questions concerning the “Healthy Eating Plate” graphic. In the first, respondents were asked to indicate its strengths. Nearly half of participants (45.0%) did not provide an opinion. Among those who responded, approximately one in six to one in five highlighted: promotion of key principles of a healthy lifestyle (19.5%), substantive content and informational value (16.0%), clarity and readability (15.0%), and appropriate design and aesthetics (13.5%).

Respondents were also asked to identify elements of the “Healthy Eating Plate” graphic that, in their view, required improvement. The vast majority of participants (83.5%) did not report any critical remarks regarding the material. The remaining comments were infrequent and concerned both graphical and substantive aspects. Some respondents noted an excessive amount of text (4.5%), an overly large emphasis on certain products (e.g., meat, fruit), and a lack of consideration for individual dietary needs (2.0% each). Additional remarks included, among others, too few illustrations (1.5%), language errors and typos (1.5%), an unclear layout, and an insufficient amount of certain products (e.g., meat) (1.0% each). Single respondents suggested including an example shopping basket with product prices (0.5%), using a smaller font size (0.5%), shortening the title (0.5%), failing to highlight important text (0.5%), removing selected components (e.g., vegetable oils) (0.5%), and the absence of an explicitly stated objective of healthy eating (0.5%).

### 3.5. Factors Determining the Level of Knowledge on Healthy Eating

The next two tables (Table 2; Table 3) present contingency matrices illustrating relationships between individual indicators of healthy eating knowledge and demographic factors (and, additionally, respondents’ self-assessed level of such knowledge). For baseline knowledge (prior to exposure to the “Healthy Eating Plate” graphic), several associations were confirmed: the overall knowledge index depended on prior use of dietetic services, and factors such as sex, age, and the aforementioned contact with a dietitian were associated with several specific aspects of knowledge (detailed information is provided later in this subsection). A statistically significant correlation was found between the level of knowledge in the pre-test and the prior self-assessment of knowledge about the principles of healthy eating (*p* < 0.05). This means that the respondents were largely able to accurately assess their level of knowledge.

Notably, in the post-test (administered after exposure to the graphic), no statistically significant associations between knowledge scores and demographic characteristics were observed (Table 3).

## 4. Discussion

This study provides, to our knowledge, the first empirical assessment of the Polish Healthy Eating Plate and its accompanying “In 3 Steps to Health” material as an educational tool for adults [5]. The pattern of results suggests that even brief exposure to a well-structured visual guide can support immediate comprehension of dietary recommendations. A plausible mechanism is that the plate format reduces cognitive load by organizing information into a small number of salient categories and by leveraging dual coding (visual structure plus short verbal cues), which may facilitate rapid recall. At the same time, the study design captures short-term knowledge transfer only; translation into sustained understanding and behaviour would likely require repeated exposure and integration into broader, multi-channel education strategies. Similarly, in a 2023 study of adults, education using the MyPlate model led to a significant increase in nutritional knowledge [7]. This result also aligns with broader evidence that structured nutrition education can improve knowledge outcomes [1,2,3].

One interpretation of the post-test pattern is that knowledge levels may have become more similar across demographic groups following exposure, although the absence of statistically significant associations should not be taken as evidence of equivalence. While baseline knowledge showed associations with prior use of dietetic services and with self-assessed knowledge, no statistically significant relationships between post-test performance and sex, age, place of residence, or educational attainment were identified. From a public health perspective, this pattern suggests that the Healthy Eating Plate may be accessible across heterogeneous segments of the adult population, potentially functioning as a low-threshold educational resource that reduces knowledge gradients related to sociodemographic factors. These findings should be interpreted as evidence of effective short-term knowledge transfer under real-world conditions rather than as proof of causal effects. When analyzing literature data on other graphic models, it can be observed that demographic differences, in the case of MyPlate, mainly concern awareness of the model’s existence, which tends to be lower in groups with a poorer socio-economic status or greater food insecurity [8]. However, in terms of willingness to use the MyPlate model, no demographic differences were found in the study by Wambagoo et al., while this report also shows that awareness of MyPlate itself varied demographically (e.g., by age, education) [9].

Post-test performance was highest for recommendations that are typically expressed as clear, action-oriented targets—namely increased whole-grain intake and reducing salt to 5 g/day (both 91.5% correct). Similarly high correct-response rates were observed for several other recommendations (e.g., plant-based fats, minimum 400 g vegetables and fruit, limiting red and processed meat, fatty fish twice weekly). These results suggest that the Healthy Eating Plate effectively communicates discrete behavioural recommendations that can be directly translated into everyday choices. Similar conclusions regarding the effectiveness of graphic communication were presented by Capitán-Gutiérrez et al., who demonstrated that the Healthy Eating Plate model not only makes it easier for recipients to identify key dietary recommendations, but also largely complies with current dietary standards for different populations, while pointing out certain limitations resulting from the simplified nature of visual tools [10].

In contrast, the most persistent deficit concerned understanding the composition of a balanced meal. Although correct responses increased from 26.0% in the pretest to 38.0% in the post-test, this remained the lowest-scoring item. Two non-mutually exclusive explanations merit consideration. First, the item format appears to have been more complex than the predominantly true/false structure used elsewhere, which may have increased cognitive load and reduced performance independent of actual comprehension. Second, this finding may reflect a genuine communication challenge and design weakness: the concept may be insufficiently salient within the visual hierarchy or may compete with other messages on the plate, leading to ambiguity about the required components of a balanced meal. This interpretation supports targeted refinements (e.g., clearer visual hierarchy, concrete meal examples, or simplified wording) and aligns with the broader view that certain concepts may require stronger scaffolding than brief exposure can provide. Some of the changes were already indicated in our previous publication [4].

Respondents’ evaluations indicated an overall positive reception of the Healthy Eating Plate, with mean ratings above the midpoint of a 7-point semantic differential scale and particularly strong ratings for comprehensibility, clarity, credibility, and attractiveness. Open-ended responses further emphasized perceived strengths related to communicating core healthy lifestyle principles and providing substantive content in a clear format. Notably, most respondents (83.5%) did not identify any elements requiring improvement, and critical comments were infrequent. Importantly, the critical comments were thematically consistent and point to actionable refinement targets: information density (too much text), editorial quality (typos), layout/visual support (too few illustrations), and perceived balance or applicability (overemphasis on selected foods; limited consideration of individual dietary needs). These qualitative signals support a user-centred refinement approach (e.g., reducing text, improving visual hierarchy, strengthening examples) and complement the quantitative findings by indicating specific design and content elements that may hinder comprehension for some users. These findings are broadly consistent with research on visual dietary benchmarks, such as MyPlate and “half-plate” rules, suggesting that simplified visual heuristics can be well understood and may support healthier self-reported choices [3].

In the present study, the combination of a plate-based visual model with brief written “Eat more/Eat less/Swap” prompts and a structured, stepwise supplementary resource may have facilitated learning through multiple channels (visual categorisation, action prompts, and staged recommendations). This approach is consistent with findings by Li et al., who demonstrated that visual nutrition education, especially when supported by additional instructional elements, improves both knowledge acquisition and healthier dietary choices [11]. Similarly, ‘Nutrition Education Linking Research, Theory, and Practice’ by Isobel R. Contento emphasizes that effective nutrition education should integrate multiple communication strategies and educational media to support the translation of knowledge into practical dietary behaviours. In this context, the multi-channel delivery used in the present study may have enhanced understanding of recommendations and supported their practical application in daily food choices [12].

An additional observation with practical relevance is that only 36.5% of respondents reported prior exposure to the Healthy Eating Plate. For comparison, during 2017–March 2020, 25.3% of American adults had heard of MyPlate [9]. This suggests that, despite being a national resource, the material’s reach may be limited and could be strengthened through wider dissemination in primary care, public institutions, workplaces, and digital channels—particularly given the high burden of overweight and obesity in Poland. From a public health practice perspective, the material’s utility likely depends less on a single exposure and more on systematic dissemination and opportunities for repeated contact (e.g., primary care, schools, workplaces, public institutions, and digital channels). In this sense, the observed response pattern can be interpreted as an argument for embedding the Healthy Eating Plate within implementation pathways that increase reach and reinforce key messages, rather than relying on stand-alone distribution.

Given the magnitude of immediate knowledge improvement and the absence of demographic disparities in post-test performance, the Healthy Eating Plate may serve as a scalable, low-cost component of broader nutrition education strategies. The material may be particularly useful where access to individual counselling is limited, complementing professional interventions rather than replacing them. Especially since access to dietitians remains limited and various concepts are emerging around the world to compensate for this shortcoming [13]. At the same time, the persistent difficulty with the balanced-meal concept points to a specific target for refinement—either within the graphic itself or through accompanying micro-interventions (e.g., short examples of “what a balanced breakfast/lunch/dinner looks like,” or interactive digital versions that support active learning). These observations align with the findings of Li et al., who found that visual nutrition education tools, particularly when combined with additional instructional supports, can enhance both knowledge acquisition and the practical application of dietary guidance, highlighting the value of multi-channel approaches in nutrition education [11].

### Limitations and Future Research

Several limitations should be considered when interpreting these findings. First, the study measured immediate post-exposure knowledge rather than long-term retention; it remains unclear whether improvements persist over time. Second, the pretest and post-test were thematically aligned but not necessarily equivalent in difficulty. In particular, the post-test relied heavily on true/false items, increasing the probability of correct responding by guessing (chance level 50%), and may therefore overestimate post-exposure knowledge and inflate apparent knowledge gains relative to more discriminating item formats. Consequently, the magnitude of improvement should be interpreted cautiously, as it may partly reflect measurement artefacts rather than genuine learning. Third, the online panel setting may limit generalisability to individuals with lower digital access or different motivational profiles. The absence of a control group limits causal inference; therefore, the observed knowledge gains cannot be unequivocally attributed to exposure to the Healthy Eating Plate alone. However, the study was designed to assess immediate knowledge transfer under real-world dissemination conditions rather than to test causal effects in an experimental framework. Fourth, the outcome assessed was knowledge, not behavioural change; although visual tools may support healthier choices, demonstrating downstream effects on dietary intake requires longitudinal or experimental designs. Fourth, the outcome assessed was knowledge, not behavioural change. Importantly, improvements in nutrition knowledge do not reliably translate into changes in dietary behaviour, which is influenced by habit formation, food availability and pricing, time constraints, cooking skills, and social-contextual factors. Consequently, these results should be interpreted as evidence of short-term knowledge transfer only, and not as proof of downstream impacts on food choice or dietary intake; establishing such effects would require controlled, longitudinal designs with behavioural endpoints.

Future studies should therefore assess retention (e.g., follow-up testing after several weeks), examine behavioural outcomes (self-reported diet quality and, where feasible, objective indicators), and compare the Healthy Eating Plate with alternative graphic models. Additionally, qualitative work could explore why the balanced-meal concept remains challenging and test targeted modifications (e.g., reduced text density, enhanced icons, culturally adapted meal examples, or stratified guidance for different dietary needs). Future evaluations should also use parallel forms matched for difficulty and item format, and consider more discriminating question types (e.g., multiple-choice with four or more options, vignette-based items, or short constructed responses) to reduce guessing effects and better capture depth of understanding.

## 5. Conclusions

This study provides pragmatic evidence of how a nationally disseminated visual nutrition guide may function as a low-burden knowledge-translation tool under conditions resembling routine public exposure. The primary contribution is methodological and public health-oriented: it demonstrates a feasible approach to rapidly test comprehension and immediate knowledge transfer for guideline graphics, while identifying a concrete communication target (the “balanced meal” concept) that appears resistant to brief exposure. These insights can inform iterative refinement of educational materials and prioritization of messages that require additional clarification or alternative formats.

At the same time, the present design does not justify strong claims of effectiveness beyond short-term knowledge acquisition. The absence of a control group, the immediate post-exposure measurement window, and item-format differences limit causal inference and do not allow conclusions about retention, behaviour change, or population-level impact. Generalizability may also be constrained by the online panel setting and by the fact that exposure occurred in a study context rather than through naturalistic, repeated contacts typical of public campaigns.

Future work should therefore test retention at meaningful follow-up intervals, include controlled comparisons (e.g., alternative graphics, text-only guidance, or no-exposure control), and extend outcomes to dietary behaviours and implementation metrics (e.g., reach, frequency of exposure, and use in primary care, workplaces, or digital channels). Mixed-methods studies could clarify why the balanced-meal message remains challenging and evaluate targeted design modifications (e.g., simplified visual hierarchy, concrete meal examples, or interactive formats) before wider rollout. Furthermore, the next step should be to update the Healthy Eating Plate model to include changes such as: adding information on portion sizes, indicating which food groups are sources of specific nutrients (e.g., whole-grain products as a source of complex carbohydrates), and including potatoes and fats in the visual representation.

## Figures and Tables

**Table 1:** Percentages of correct responses to questions assessing knowledge of healthy eating (prior and after to exposure to the Healthy Eating Plate graphic) (n = 200).

Area of Knowledge Prior to Exposure to the Healthy Eating Plate Graphic	% SD	Area of Knowledge After to Exposure to the Healthy Eating Plate Graphic	%
Vegetables and/or fruit should be consumed with every meal.	85.0% ±35.7%	An important component of healthy eating guidelines is consuming at least 400 g of a variety of colourful vegetables and fruit per day.	88.5% ±31.9%
Fish, particularly fatty marine fish, are an important component of a healthy diet.	83.5% ±37.1%	It is recommended to aim to consume a variety of fatty fish twice per week.	87.0% ±33.6%
Red meat and processed meat products should be replaced with fish, poultry, eggs, legumes, and nuts.	82.5% ±38.0%	It is recommended to replace meat with plant-based protein sources (e.g., legumes and nuts), as well as with fish and eggs.	86.5% ±34.2%
Increasing the proportion of legumes in the diet is beneficial to health.	82.5% ±38.0%	Consumption of red meat and processed meat products should be limited to 500 g per week.	87.5% ±33.1%
A healthy diet is characterized by a higher intake of whole-grain products than refined ones.	76.0% ±42.7%	One of the key recommendations is to consume more whole-grain products.	91.5% ±27.9%
Milk should not be consumed only by children and adolescents.	73.0% ±44.4%	The recommended daily serving of dairy is two glasses of milk, which may be replaced with yoghurt, kefir, buttermilk, or cottage cheese.	80.0% ±40.0%
Canola oil is a healthy product worth using in meal preparation.	64.0% ±48.5%	It is recommended to replace animal-derived fats with plant-based fats.	89.5% ±30.7%
Intake of low-fat milk and dairy products, particularly fermented products, should be increased.	57.0% ±49.5%	Vegetables should account for a greater share of the diet than fruit.	81.0% ±39.2%
Salt intake should not be eliminated completely.	51.5% ±50.0%	Salt intake should be reduced to 5 g (one teaspoon) per day.	91.5% ±27.9%
A balanced meal consists of vegetables or fruit, a protein source, whole-grain products, and fat.	26.0% ±43.9%	A balanced meal consists of vegetables or fruit, a protein source, whole-grain products, and fat.	38.0% ±48.5%
Consuming whole-grain products once per day is not sufficient.	26.0% ±43.9%	The recommended intake of whole-grain products is three servings per day.	70.5% ±45.6%

**Table 2:** Correlation coefficients between socio-geographical factors and knowledge about healthy eating (prior to exposure to the Healthy Eating Plate graphic) (n = 200).

	Sex	Age	Type of Residence	Education Level	Self-Assessed Knowledge of Healthy Eating	Previous Use of Dietetic Services
Overall index of knowledge on healthy eating (prior to exposure to the Healthy Eating Plate graphic)	ρ = −0.135, *p* > 0.05	ρ = 0.025, *p* > 0.05	ρ = −0.013, *p* > 0.05	ρ = 0.059, *p* > 0.05	ρ = 0.0294, *p* < 0.05	ρ = 0.128, *p* < 0.05
Vegetables and/or fruit should be consumed with every meal	ρ = 0.028, *p* > 0.05	ρ = 0.064, *p* > 0.05	ρ = −0.076, *p* > 0.05	ρ = −0.05, *p* > 0.05	ρ = 0.046, *p* > 0.05	ρ = −0.026, *p* > 0.05
Fish, particularly fatty marine fish, are an important component of a healthy diet.	ρ = −0.014, *p* > 0.05	ρ = −0.016, *p* < 0.05	ρ = −0.043, *p* > 0.05	ρ = 0.053, *p* > 0.05	ρ = 0.052, *p* > 0.05	ρ = −0.049, *p* > 0.05
Red meat and processed meat products should be replaced with fish, poultry, eggs, legumes, and nuts	ρ = −0.04, *p* > 0.05	ρ = −0.055, *p* < 0.05	ρ = −0.071, *p* > 0.05	ρ = 0.01, *p* > 0.05	ρ = 0.154, *p* > 0.05	ρ = 0.053, *p* > 0.05
Increasing the proportion of legumes in the diet is beneficial to health	ρ = 0.04, *p* > 0.05	ρ = 0.054, *p* > 0.05	ρ = −0.043, *p* > 0.05	ρ = 0.092, *p* > 0.05	ρ = 0.042, *p* < 0.05	ρ = −0.001, *p* > 0.05
A healthy diet is characterized by a higher intake of whole-grain products than refined ones	ρ = 0.047, *p* > 0.05	ρ = 0.174, *p* > 0.05	ρ = 0.099, *p* > 0.05	ρ = 0.104, *p* > 0.05	ρ = 0.132, *p* > 0.05	ρ = −0.006, *p* > 0.05
Milk should not be consumed only by children and adolescents	ρ = −0.18, *p* > 0.05	ρ = 0.009, *p* > 0.05	ρ = −0.021, *p* > 0.05	ρ = −0.037, *p* > 0.05	ρ = 0.135 *p* < 0.05	ρ = 0.046, *p* > 0.05
Canola oil is a healthy product worth using in meal preparation	ρ = −0.104, *p* < 0.05	ρ = 0.184, *p* > 0.05	ρ = −0.065, *p* > 0.05	ρ = 0.053, *p* > 0.05	ρ = 0.082, *p* > 0.05	ρ = 0.014, *p* > 0.05
Intake of low-fat milk and dairy products, particularly fermented products, should be increased	ρ = −0.061, *p* > 0.05	ρ = −0.009, *p* > 0.05	ρ = 0.022, *p* > 0.05	ρ = 0.052, *p* > 0.05	ρ = 0.193, *p* < 0.05	ρ = 0.164, *p* < 0.05
Salt intake should not be eliminated completely	ρ = −0.09, *p* > 0.05	ρ = −0.121, *p* > 0.05	ρ = 0.052, *p* > 0.05	ρ = 0.035, *p* > 0.05	ρ = 0.112, *p* > 0.05	ρ = 0.101, *p* > 0.05
A balanced meal consists of vegetables or fruit, a protein source, whole-grain products, and fat	ρ = −0.046, *p* > 0.05	ρ = 0.025, *p* > 0.05	ρ = 0.001, *p* > 0.05	ρ = 0.03, *p* > 0.05	ρ = 0.115, *p* > 0.05	ρ = 0.089, *p* > 0.05
Consuming whole-grain products once per day is not sufficient	ρ = −0.137, *p* > 0.05	ρ = −0.138, *p* < 0.05	ρ = −0.088, *p* > 0.05	ρ = −0.041, *p* > 0.05	ρ = 0.143, *p* > 0.05	ρ = 0.112, *p* > 0.05

**Table 3:** Correlation coefficients between socio-geographical factors and knowledge about healthy eating (after exposure to the Healthy Eating Plate graphic) (n = 200).

	Sex	Age	Type of Residence	Education Level	Self-Assessed Knowledge of Healthy Eating	Previous Use of Dietetic Services
Overall index of knowledge on healthy eating (after exposure to the Healthy Eating Plate graphic)	ρ = −0.026, *p* > 0.05	ρ = 0.056, *p* > 0.05	ρ = −0.065, *p* > 0.05	ρ = 0.048, *p* > 0.05	ρ = 0.048, *p* > 0.05	ρ = −0.026, *p* > 0.05
One of the key recommendations is to consume more whole-grain products	ρ = 0.054, *p* > 0.05	ρ = 0.024, *p* > 0.05	ρ = −0.094, *p* > 0.05	ρ = 0.07, *p* > 0.05	ρ = −0.086, *p* > 0.05	ρ = 0.054, *p* > 0.05
Salt intake should be reduced to 5 g (one teaspoon) per day	ρ = −0.018, *p* > 0.05	ρ = 0.1, *p* > 0.05	ρ = 0.02, *p* > 0.05	ρ = 0.07, *p* > 0.05	ρ = 0.044, *p* > 0.05	ρ = −0.018, *p* > 0.05
It is recommended to replace animal-derived fats with plant-based fats	ρ = −0.049, *p* > 0.05	ρ = 0.073, *p* > 0.05	ρ = −0.03, *p* > 0.05	ρ = 0.028, *p* > 0.05	ρ = 0.047, *p* > 0.05	ρ = −0.049, *p* > 0.05
An important component of healthy eating guidelines is consuming at least 400 g of a variety of colourful vegetables and fruit per day	ρ = 0.11, *p* > 0.05	ρ = 0.036, *p* > 0.05	ρ = −0.057, *p* > 0.05	ρ = 0.001, *p* > 0.05	ρ = 0.049, *p* > 0.05	ρ = 0.11, *p* > 0.05
Consumption of red meat and processed meat products should be limited to 500 g per week	ρ = 0.106, *p* > 0.05	ρ = 0.039, *p* > 0.05	ρ = −0.125, *p* > 0.05	ρ = −0.024, *p* > 0.05	ρ = 0.032, *p* > 0.05	ρ = 0.106, *p* > 0.05
It is recommended to aim to consume a variety of fatty fish twice per week	ρ = −0.06, *p* > 0.05	ρ = 0.072, *p* > 0.05	ρ = −0.089, *p* > 0.05	ρ = −0.035, *p* > 0.05	ρ = −0.03, *p* > 0.05	ρ = −0.06, *p* > 0.05
It is recommended to replace meat with plant-based protein sources (i.e., legumes and nuts), as well as with fish and eggs	ρ = 0.015, *p* > 0.05	ρ = 0.067, *p* > 0.05	ρ = −0.075, *p* > 0.05	ρ = −0.062, *p* > 0.05	ρ = −0.001, *p* > 0.05	ρ = 0.015, *p* > 0.05
Vegetables should account for a greater share of the diet than fruit	ρ = −0.051, *p* > 0.05	ρ = −0.063, *p* > 0.05	ρ = 0.072, *p* > 0.05	ρ = 0.045, *p* > 0.05	ρ = 0.084, *p* > 0.05	ρ = −0.051, *p* > 0.05
The recommended daily serving of dairy is two glasses of milk, which may be replaced with yoghurt, kefir, buttermilk, or cottage cheese	ρ = −0.025, *p* > 0.05	ρ = 0.025, *p* > 0.05	ρ = −0.058, *p* > 0.05	ρ = 0.089, *p* > 0.05	ρ = 0.1, *p* > 0.05	ρ = −0.025, *p* > 0.05
The recommended intake of whole-grain products is three servings per day	ρ = −0.033, *p* > 0.05	ρ = 0.022, *p* > 0.05	ρ = −0.005, *p* > 0.05	ρ = 0.046, *p* > 0.05	ρ = −0.029, *p* > 0.05	ρ = −0.033, *p* > 0.05
A balanced meal consists of vegetables or fruit, a protein source, whole-grain products, and fat	ρ = −0.124, *p* > 0.05	ρ = −0.009, *p* > 0.05	ρ = 0.008 *p* > 0.05	ρ = 0.046, *p* > 0.05	ρ = 0.052, *p* > 0.05	ρ = −0.124, *p* > 0.05

## Data Availability

The original contributions presented in this study are included in the article Further inquiries can be directed to the corresponding author(s).
